# Pairwise causal discovery in biochemical networks: A survey on directionality inference within complex networks from stationary observations

**DOI:** 10.1371/journal.pone.0349617

**Published:** 2026-06-16

**Authors:** Nava Leibovich, Miroslava Cuperlovic-Culf

**Affiliations:** 1 National Research Council of Canada, NRC-Fields Mathematical Sciences Collaboration Centre, Toronto, Ontario, Canada; 2 Digital Technologies Research Centre, National Research Council Canada, Ottawa, Ontario, Canada; 3 Department of Biochemistry, Microbiology and Immunology, Faculty of Medicine University of Ottawa, Ottawa, Canada; 4 Ottawa Institute of Systems Biology, University of Ottawa, Ottawa, Canada; Louisiana State University Health Sciences Center, UNITED STATES OF AMERICA

## Abstract

Metabolic networks map complex biochemical reactions within organisms, which is crucial for understanding cellular processes and metabolite flow. This study focuses on inferring the directionality of interactions in metabolomics networks. Given the challenge of using steady-state data, we benchmark various methods, including statistical scores and neural network approaches, on synthetic yet realistic biological models. Our findings highlight the relative success of a few methods in some cases where the interaction mechanism is known, whereas other methods show limited effectiveness.

## Introduction

Metabolic networks function as intricate maps of biochemical reactions within an organism, illustrating the complex web of interactions that govern cellular processes. Understanding the directionality of these reactions is crucial for tracking the flow of metabolites through pathways and illuminating the functional dynamics of cellular metabolism. This information is relevant across various fields, including systems biology, biotechnology, and drug discovery [1–5]. In this context, we focus on the interpretation of the metabolomics network.

The metabolome consists of hundreds of correlated compounds, offering substantial information within the metabolomics network that extends beyond the mere levels of individual metabolites [6–8]. Focusing solely on individual metabolites can obscure potential intervention targets due to collinearity and confounding factors; however, network models can effectively incorporate these interrelationships within the metabolome [6–10]. The significance of biological networks in human diseases has been widely acknowledged [11,12], particularly in their role in the identification of causal associations [13].

Interaction networks are typically inferred using time series measurements or pseudo-time trajectories, employing statistical tools such as Bayesian inference and maximum likelihood alongside machine learning algorithms [14–24]. Notably, a wide range of recent studies have explored methods for inferring networks from temporal data, as highlighted in the partial list as follows [25–35]. However, it is important to note that these methods require the records of synchronized ordered data, which is often unmeasurable in various observational contexts.

**Fig 1:**
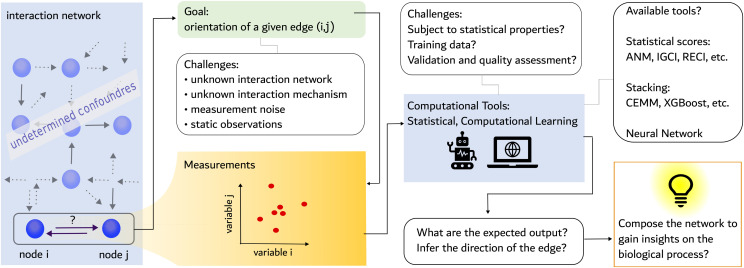
The main goal is to infer the direction of interaction between two variables within a metabolic network. Our focus is on methods designed to uncover the directional influence between these two variables. However, determining the orientation of the network from static data poses a great challenge, necessitating keen analytical and computational techniques.

Here, we aim to infer the direction of interactions from stationary data associated with the analyzed dataset, see Fig 1. In general, the interaction network can be curated from prior biological knowledge, or structured from experimental data [36–39]. The experimental networks are directly derived from data, for example, untargeted metabolomics generated from mass spectrometry [36–39]. One can derive the *functional* connectivity, which provides valuable insights into the statistical dependencies arising from the collective dynamics of interactions between pairs of units [40–46]. These statistical dependencies among components can be characterized by well-known measures such as correlation, mutual information, or their analyses using silencing methods [44–48]. However, these measures are symmetric and consequently do not capture the direction of interactions, resulting in the inference of non-directed networks. In contrast, one may consider non-symmetric metrics, such as partial correlations or dependency analyses. Specifically, partial correlation and various kernel-based metrics have been proposed to analyze associations between variables while accounting for confounders within the functional connectivity network [49–54]. However, these approaches require recording data from all other interacting variables in the network, including any suspected confounders. Such comprehensive data is often unavailable in many systems.

In particular, we recognize that the complete metabolic network under consideration has not been fully characterized. As a result, neither the overall network topology nor other confounding variables have been adequately described or measured. Consequently, we focus on methods designed to infer the pairwise causal direction between two correlated variables. Furthermore, we emphasize that earlier benchmark analyzes of pairwise inference methods [55–57] evaluate performance with respect to various causal mechanisms between pairs of variables, without taking into account the context of interaction networks in biological processes. Such networks can introduce intricate influences due to signal propagation, even when the two variables do not directly interact with one another.

Determining the network orientation from steady-state data is therefore challenging. First, we examine various methods for deciphering interaction directions using steady-state data and highlight their domains of failure and success. We survey both statistical scores, which allow inference from small data sets, and methods based on machine learning and neural network approaches, which require large data sets for training the models. We tested these methods on multiple interaction mechanisms, with and without additional noise.

## Main results

As mentioned, our aim is to infer the direction of interaction within a partially observed interaction network between biochemical components to gain insights into some biological processes. To do so, we benchmark existing methods to infer directionality from pairwise data. Our contribution lies in the following. Previous comparisons between methods collate various interaction mechanisms, combining linear and non-linear effects, and additive and multiplicative noise. Nevertheless, these previously examined mechanisms do not necessarily capture the complexity of biochemical reaction networks; which possess multiple confounders that may remain unknown, including biochemical cycles and interaction loops, complex signal propagation throughout the network, and which the pairwise interactions mechanism is unknown.

### Benchmarking methods for analyzing synthetic data

We use several interaction mechanisms within complex networks. Specifically, we use the Michaelis-Menten model, which describes gene interaction networks [58–60], as well as networks of coupled Rössler oscillators, and coupled Goodwin oscillators, which represent a prototypical biological oscillator that characterizes various biological processes such as circadian clocks [61–65], and brain function [66]. These synthetic models are commonly used in literature for describing biological processes. More details on these models can be found in S1 Appendix.

Generally, the investigated interactions can be derived from either knowledge-based or experimental networks [36–39]. In our synthetic data, all examined edges correspond to mathematically defined interactions, which means they are knowledge-based reactions. We analyze both random Erdős–Rényi networks, characterized by a binomial degree distribution, and Barabási–Albert scale-free networks, which exhibit a power-law degree distribution. We start with considering the Erdős–Rényi networks, where the results for Barabási–Albert networks are given in Subsect. Network structure dependence: Scale-Free vs Erdős–Rényi Models below. Examination of these two network models enables a comprehensive evaluation of network-dependent behaviors relevant to our assessment.

**Fig 2:**
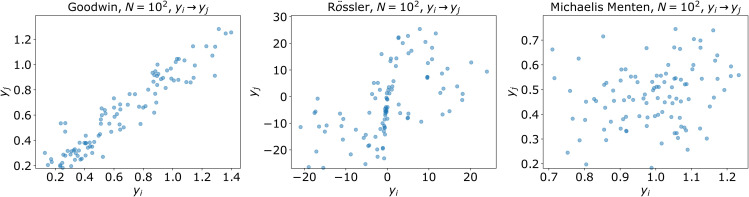
Realizations of interacting variables’ pairs y_i_, y_j_ for different interaction mechanisms; Goodwin, Rössler, and Michaelis-Menten models (left to right correspondingly). For consistent visualization, we present the direction of interaction y_i_ → y_j_ for all panels, where for each we sample N=10^2^ data points.

First, we consider random interaction networks following the Erdös–Rényi model. In Fig 2 we show the observations of pairs for the interaction mechanisms considered. Given that both variables are affected by additional influences from other network variables, along with inherent noise for each variable, which also propagates throughout the network, determining the direction of the interaction is highly challenging [67].

We investigate a range of methods, including those grounded in statistical scores, stacking techniques, and artificial neural networks (NNs). The statistical scores we analyzed include the Additive Noise Model (ANM), Conditional Distribution Similarity (CDS), Information Geometric Causal Inference (IGCI), and Regression-Error-based Causal Inference (RECI). Additionally, we examined various stacking methods, such as voting, eXtreme Gradient Boosting (XGBoost), and a recently introduced Causal Ensemble Learning method based on Support Measure Machines (CEMM). Furthermore, we explore several proposed deep neural network architectures, such as the Neural Causation Coefficient (NCC) and Randomize Causation Coefficient (RCC). A comprehensive overview and description of these inference methods can be found in Table 1 and in S1 Appendix. The implementations were based on [68] wherever applicable.

**Table 1:** Pairwise interaction orientation methods.

Methods [Ref.]	Learning approach			Data type				comments
	statistical	NN ^*b*^	ensemble	continuous	categorical	mixed	discrete	
Additional Noise Model [79,80]	X			X				assume additive noise; y=f(x)+η ^*a*^
Bi-variate fit [56]	X			X				Gaussian process regression
Conditional Distribution Similarly Statistics [81]	X			X			X	
Information Geometric Causal Inference [82]	X			X				*y*=*f*(*x*) with *f* inevitable ^*a*^
Revealing interaction from collective dynamics [65]	X			X				uses system response to known driving signals
Regression Error based Causal Inference [83]	X			X				
Sensitivity Analyses of Reaction Rates [84]	X						X	assume self-regulation is known
Shallow Generated Neural Network [85]		X		X				DAG, model NN with 1 hidden layer between x and y, and between y and x
Neural Causation Coefficient [86]		X		X	X	X		
Randomize Causation Coefficient [87]		X		X	X	X		projection of RKHS using random cosine embedding ^*b*^
Convolutional Neural Network [88]		X		X	X	X	X	
JARFo [81]			X	X	X	X		stacking statistical features with gradient boosting classification
stacking via voting			X	X				stacking statistical scores with voting
XGBoost stacking			X	X				stacking statistical scores using XGBoost ^*b*^
Meta-Learning using FiLM layer ^*b*^ [89]			X	X				meta-data learning using encoder-decoder architecture
Causal Ensemble Measure Machine [56]			X	X				stacking statistical scores using SMM ^*b*^

*a* For uniform notation we assume that the variable *x* affects the variable *y*, i.e., x→y. The noise term is marked with η.

*b* Abbreviations: NN = neural networks, RHHS = reproducing kernel Hilbert space, DAG = directed acyclic graph, FiLM = Feature-wise Linear Modulation, XGBoost = eXtreme Gradient Boosting, SMM = Support Measure Machine.

The techniques that have been tested provide a rich composition of methods, each incorporating different approaches to inference. This variety offers a solid foundation for benchmarking assessment. However, we have not examined all possible methods that have been proposed in the literature. Certain methods were excluded for several reasons, including limitations in computational capacity, the unavailability of published code from the relevant articles, and a lack of applicability to our data. Specifically, some methods require information that is inaccessible or involve data types that do not align with the data of our interest. As mentioned, the methods are reviewed in Table 1 and S1 Appendix.

### Performance on random synthetic networks

**Fig 3:**
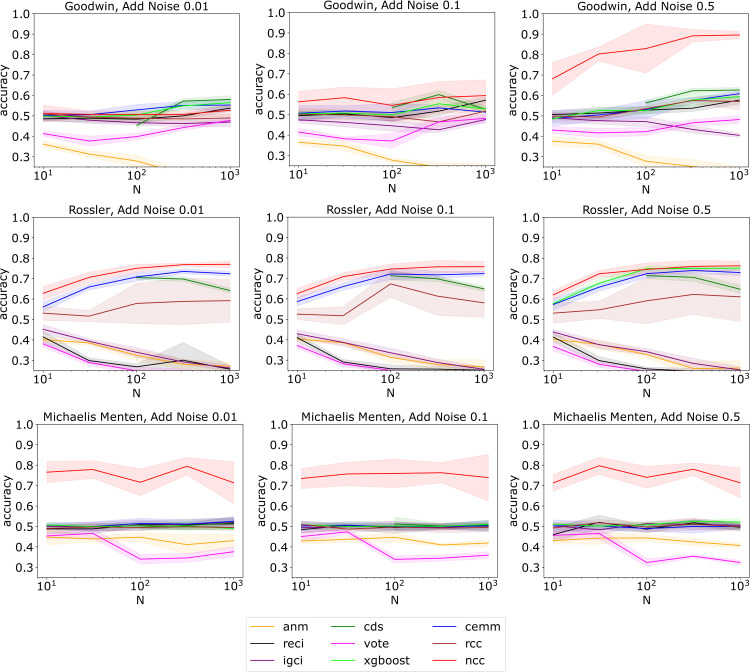
Results for the tested directionality inference methods for various interaction mechanisms. Here the interaction networks are from Erdős–Rényi type.

We show in Fig 3 that for the biochemical reaction networks we examined, most of the methods we assessed struggle with direction determination, as no method presents excellent accuracy, say beyond 0.9. This somewhat inferior accuracy is expected, especially for the statistical scores, since they rely on strong assumptions about the statistical characteristics of the data, which are not necessarily complied with within our systems. In general, an accuracy of around 0.5 indicates a random choice between two equally probable cases, while an accuracy lower than that implies systematic errors in the statistical scores from their unfulfilled assumptions.

However, we found that some methods perform better than others. In particular, the NCC method yields results that surpass the baseline of random choice located at 0.5, with accuracies ranging between 0.7 and 0.9. We find that the NCC results exceed other methods for various interaction mechanisms and noise scales that were considered. We further investigate insights from these results in the following subsections.

Interestingly, for the coupled Rössler oscillators, we found that the CEMM, XGBoost, and CDS methods produced relatively successful results, albeit falling short compared to the NCC method (see Fig 3). Among stacking methods, XGBoost and CEMM demonstrated accuracy that places them second to NCC, achieving an approximate accuracy of 0.7. Additionally, for the Rössler coupled oscillators, the statistical score of CDS exhibits similar accuracy for larger values of *N*. In contrast, for the other interaction mechanisms that were examined, the CDS, CEMM, and XGBoost stacking methods, along with the other inference approaches tested—excluding NCC—failed to accurately infer the direction of the interaction.

In addition, our findings indicate some dependence on the noise level. We tested the performance for three noise levels. For data synthesized with the Goodwin mechanism, a higher noise level seems preferable, and success was found for the NCC method. Other dynamic mechanisms show comparable results for all noise levels tested. Additionally, the finite number of sampled data points, *N*, induced additional measurement noise from the finite sampling itself. For small *N*, most methods show an accuracy close to 0.5, which signifies the chance accuracy of a dummy classifier for the two possible directions. As *N* increases, some methods improve their accuracy, while for others the accuracy remains at the random chance or even decreases below it, due to systematic errors that emerged from the statistical assumption. Given that observations are typically of the size of $10^{2}-10^{3}$ samples, we do not consider a larger set of data points. Furthermore, we examine the results when varying the level of missing data using imputation. We have tested multiple imputation methods: replacement of the missing values with the mean, iteratively predicting missing values using the MICE (Multiple Imputation by Chained Equations) method [69], and the imputation using the K-Nearest Neighbors (KNN) approach [70]. The dependence of varying the rate of missing values on the accuracy of the methods for inference of the interactions’ direction shows similar results, where the accuracy of NCC model exceeds the random classifier results, see S1 Appendix.

### Further inspection of the NCC model

**Fig 4:**
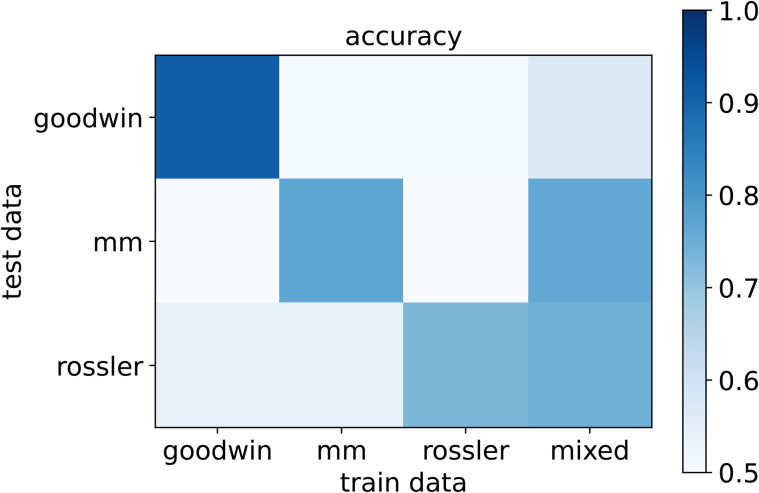
Testing data vs trained NCC models. As expected, using training and testing datasets from different mechanisms is ineffective.

To expand the analyses even further, we have tested the NCC method as a candidate for a directionality inference method. We first examine the performance of the model trained on a given dataset drawn from one mechanism, to infer interaction direction from another mechanism, see Fig 4. As expected, each NCC model performs better on the data mechanism it was trained on. Obviously, for a trained model to be effective, the training data must be similar to the data aimed to infer. Nevertheless, in the biological processes under question, one does not necessarily have such information.

We also trained an additional model using simulated data generated with randomly selected interaction mechanisms, referred to as “mixed.” Unlike before, we did not specify particular mechanisms. This new model yielded slightly improved results, exceeding the random accuracy for two different mechanisms we examined.

**Fig 5:**
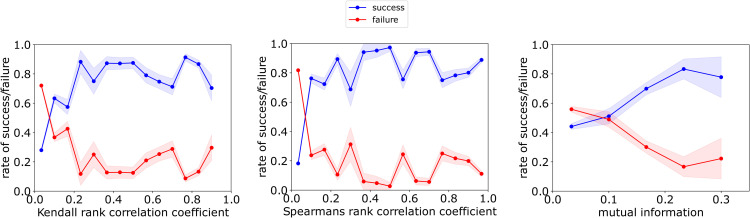
Dependence quantities influence the probability for meaningful training of the NCC model and thus to a successful inference. Results for the Kendall τ coefficient (left), Spearman ρ coefficient (middle), and mutual information (right) show that stronger dependence between the two variables corresponds to increased success rates in determining the direction of interaction between them.

The analysis considers Kendall’s τ and Spearman’s ρ correlation coefficients, as well as the mutual information between variables, to evaluate their observed dependence. Higher values of these metrics indicate that the dynamics of one variable are influenced by the dynamics of the other. Our findings show that a strong dependence is associated with a higher probability of correctly determining the direction of interaction, as shown in Fig 5. In our synthetic data, all examined edges correspond to defined interactions in which one variable categorically influences the other. In other words, we consider the edge to be given from prior knowledge on the process [36–38]. Nevertheless, weak interactions may be obtained empirically due to sampling errors, noise propagation from other network regions, or confounding factors affecting both variables. Fig 5 shows that edges with weakly measured interactions are less likely to be inferred correctly. Most of these interactions, however, would have likely been omitted in an experimental network since their pairwise dependence would be lower than the required threshold. As was previously noted, integration of knowledge-based networks with experimental networks would help to improve the identification and interpretation in biologically relevant contexts [36–39].

### Network structure dependence: Scale-Free vs Erdös–Rényi models

**Fig 6:**
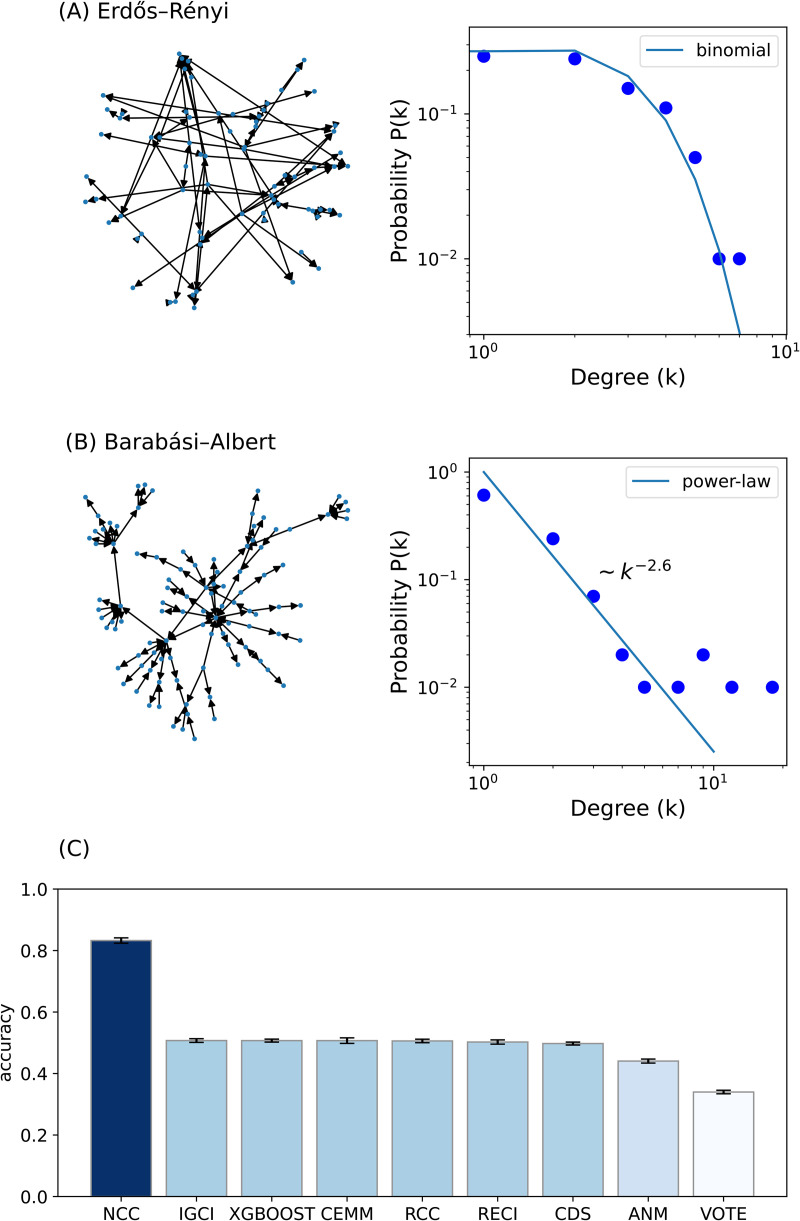
Comparison between Erdös–Rényi and Barabási–Albert network structures and corresponding inference performance. (A) Example of a random network generated using the Erdös–Rényi model and its degree distribution, which follows a binomial pattern. (B) Example of a scale-free network generated using the Barabási–Albert model, exhibiting a power-law degree distribution. Both networks share the same mean degree but display markedly different topologies. (C) Performance of directional inference methods applied to reactions (edges) drawn from scale-free interaction networks. Simulations were performed using Michaelis–Menten dynamics with a noise scale of 0.5 and a dataset size of N = 10^3^.

Up to this point, we have presented results for edges within random Erdös–Rényi-type networks. However, scale-free networks are frequently reported in complex biological systems [71–74]. A key property of these networks is their degree distribution, which follows a power-law pattern, $P(k)\sim k^{-\gamma }$ where *k* is the degree and the exponent γ typically ranges between 2 and 3. Fig 6 compares random networks generated using the Erdös–Rényi model (Fig 6A) and the Barabási–Albert scale-free model (Fig 6B). Although both networks have the same mean degree, their topologies differ substantially. In particular, Barabási–Albert network exhibits ‘hubs’ where some nodes have high degrees, while the vast majority of nodes have a degree of one and, as such, are connected to only a single node. Fig 6C shows the performance of directional inference methods for interactions governed by Michaelis–Menten dynamics, where nodes represent variables in scale-free networks. The results show a consistent pattern: among the tested methods, the NCC approach demonstrates superior performance over the others.

### Model performance for realistic biological systems

We test the applicability of the above models for inference of the direction of the interaction within realistic biological processes. To do so, we simulate these biological processes and test the performance of the inferred interaction direction. Specifically, we first use a previously proposed biologically realistic system – a model that is based on the *E. coli* gene regulatory network (GNR) provided in the DREAM challenge [75–77]. In addition, we simulate the Bile Acid Synthesis Pathway (BASP) model specified in [78] to describe a part of cholesterol metabolism. Finally, we simulate sphingolipid metabolism using computationally determined enzyme kinetic parameters and a detailed metabolic network based on KEGG: map00600. The details of all models are provided in the S1 Appendix.

**Fig 7:**
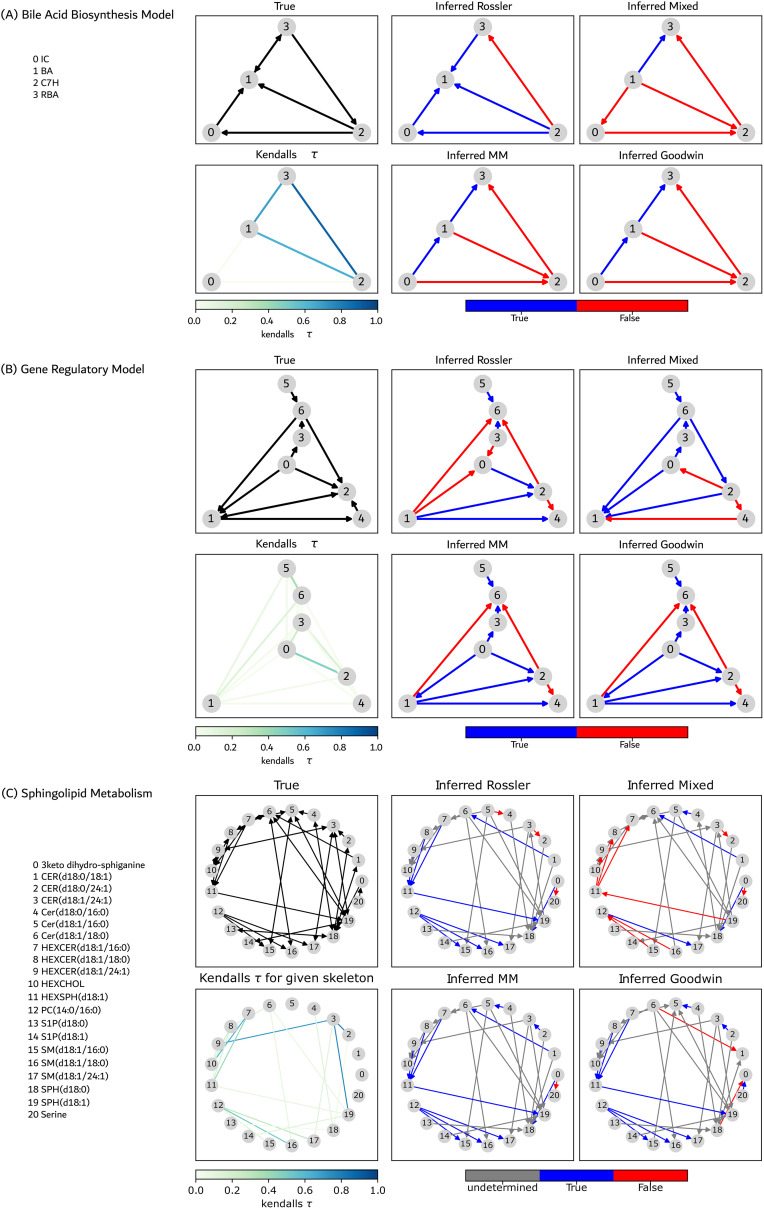
Performance for Realistic Biological Systems. The models used for direction inference are NCC with training data as specified in each panel. Models are detailed in (S1 Appendix).

Testing the models on synthetic, yet realistic, biological process data enables us to evaluate the anticipated performance of the inference method in a controlled environment before applying it to actual data of interest. We utilize our pre-trained NCC models and apply them to the biological processes mentioned above, see Fig 7. Our simulation results indicate that the inference approaches explored demonstrate limited success in elucidating the interactions within the examined biological network.

## Discussion and summary

Inferring the direction of interaction between two components within a complex biochemical network from time-independent observations is an important yet challenging task. One key significance of inferring directed biochemical networks in human health research lies in the ability to control and recover metabolic performance in faulty or suboptimally operating cells, such as those with mutations [5]. Additionally, it is essential to understand how signal transduction networks influence cancer cell behavior, including proliferation, survival, invasiveness, and drug resistance [3]. Therefore, the study of biological interaction networks, and in particular the inference of the direction of biochemical reactions, is essential as it may eventually lead to new therapeutic approaches and personalized treatments.

We examined various methods to determine the directionality of the interaction while considering the characteristics of the biological observations of metabolites; the small data set, many variables that are unknown or unobserved, the multiplicity of confounders, the propagation of noise through the network, the unknown dynamics mechanism between the variables, and more. Since many variables are undetermined, we focused on pairwise directionality inference. The directionality inference approaches discussed in the paper focus on determining a single direction between a pair of variables. We note that the analysis excludes bidirectional edges, which describe reversible reactions. Considering this type of interaction requires expansion of the methods beyond their current capacity of binary classification of direction. Further examination of whether the raw scores for each direction, or other computational approaches, may be utilized to distinguish reversible reactions is left for future work.

We compared various approaches and found that they are limited in determining the direction of influence effectively. This limitation may stem from unfulfilled assumptions, such as the presence of directed acyclic graphs or the introduction of additional noise. Furthermore, complexity can increase due to different interaction mechanisms, multiple undetermined confounders, and various sources of noise. Therefore, we suggest that methods for determining the orientation of interaction should be tailored specifically to the system being studied whenever possible.

The inference of pairwise directionality from multiple snapshots, especially in the absence of temporal information, should be approached with caution. In particular, one must be wary of interpreting the interaction directionality as indicative of ’causality.’ This term refers to scenarios where direct changes or interventions in one variable influence the state of another variable. Consequently, ’causality’ is typically discussed in the context of temporal data, where direct interventions and their subsequent effects can be observed over time. However, while some argue that ’causality’ should only apply to temporal data, others extend its definition to include an interpretation based on static observations – this dissension remains open for further research.

## Supporting information

S1 Appendix Appendices. (PDF)
