# Supplementary material for: Pairwise causal discovery in biochemical networks: A survey on directionality inference within complex networks from stationary observations
Source: PLoS One. 2026 Jun 16;21(6):e0349617. doi: 10.1371/journal.pone.0349617 (PMC13271444; doi:10.1371/journal.pone.0349617)
Supplement: S1 Appendix — (PDF) [file pone.0349617.s001.pdf]

SUPPLEMENTARY INFORMATION

Appendix A: Synthetic Data generation

For numerical demonstrations, we generate a random directed graph $\hat{G}$ that holds the topology of the network. Mathematically, the graph is described by the adjacency matrix with elements $G_{ij} = 1$ where the state of j affects the dynamics of node i, i.e., $j \to i$, and $G_{ij} = 0$ otherwise. We examine systems with Erdös Rényi random networks similarly to [1].

The synthetic data is generated as follows. First, we generate a long temporal trajectory of (y_i, y_j) following

$$\partial_t y_i(t) = \sum_j G_{ij} \times \mathrm{Int}[y_i(t), y_j(t)] + \text{noise}. \tag{A1}$$

Here, $\hat{G}$ is the adjacency matrix of the Erdös Rényi random interaction network. The function $\mathrm{Int}[y_i, y_j]$ defines both the mechanism of the interaction, the effect of variable $j \neq i$ on the dynamics of variable i, and the self-regulation, the effect of the level y_i on the dynamics of oneself. The noise term can be either additive noise or multiplicative noise.

We note that we do not aim to determine the interaction mechanism $\mathrm{Int}[y_i, y_j]$ itself, and we do not subject ourselves to any specific form of it. Moreover, we note that $\mathrm{Int}[y_i, y_j]$ defines only direct interaction between the variables. However, indirect effects might present as well, especially in cases where the networks we examine might be cyclic graphs due to the nature of the biochemical process.

1. Models

Continuum Michaelis–Menten Regulatory Network

The dynamic in the Michaelis–Menten (MM) regulatory network is given by the

$$\partial_t y_i(t) = \sum_{j \neq i} G_{ij} \times f[y_i(t), y_j(t)] - \beta_i y_i + \text{noise}, \tag{A2}$$

where the interaction function for a given edge is given by

$$f[y_i(t), y_j(t)] = y_j(t)/[K + y_j(t)]$$
$$\text{or} \tag{A3}$$
$$f[y_i(t), y_j(t)] = K/[K + y_j(t)]$$

where we choose between the activation (top) and suppression (bottom) randomly, with equal probability of either choice. For variables that are not affected by any other variable, i.e., $\sum_j G_{ij} = 0$, we use; $f = 1$. The argument of the half-maximum rate is determined as $K = 1$. All variables are self-regularized by the term $-\beta_i y_i$, where β_i represents the degradation rate of molecule i and is chosen in the simulation to be drawn from Gaussian distribution with mean 1 and noise scale of 0.1.

Michaelis–Menten kinetics describes enzyme-catalyzed reactions in which a substrate (S) is converted into a product (P) by an enzyme (E). The overall process can be represented as $S + E \rightleftharpoons ES \rightarrow E + P$. Under steady-state conditions, the reaction rate is expressed as $\partial_t[P] = V_{\max}[S]/(K_m + [S])$, where $V_{\max}$ is the maximum reaction rate and K_m is the substrate concentration at which the rate is half of $V_{\max}$.

Coupled Goodwin Oscillators

The dynamic for the triplet $(x_i(t), y_i(t), z_i(t))$ is given by the

$$\begin{aligned}
\partial_t x_i(t) &= \frac{v_0}{1 + (z_i(t)/K)^n} - a_i x_i(t) + \text{noise} \\
\partial_t y_i(t) &= x_i(t) - b_i y_i(t) - \sum_i^N G_{ij} \times (y_i - y_j) + \text{noise} \\
\partial_t z_i(t) &= y_i(t) - c_i z_i(t) + \text{noise}
\end{aligned} \tag{A4}$$

where the numeric simulation took place with the parameters $K = 1$, $a_i = b_i = c_i = 0.4$, $v_o = 1$, $n = 17$ the same as in [1].

Rössler Oscillators

The dynamic for the triplet $(x_i(t), y_i(t), z_i(t))$ is given by the

$$\begin{aligned}
\partial_t x_i(t) &= y_i(t) + a_i x_i(t) + \text{noise} \\
\partial_t y_i(t) &= -x_i(t) - z_i(t) + \sum_i^N G_{ij} \times \sin(y_j) + \text{noise} \\
\partial_t z_i(t) &= b_i + z_i(t) \times y_i(t) - c_i z_i(t) + \text{noise}
\end{aligned} \tag{A5}$$

where the numeric simulation took place with the parameters $K = 1$, $a_i = 0.1$, $b_i = 1$ and $c_i = 18.0$ for every i as the same as [2].

Bile Acid Synthesis Pathway

The Bile Acid Synthesis Pathway (BASP) is modeled by

$$
\begin{aligned}
\partial_t x_0(t) &= D - \frac{k_3 x_2(t) x_0(t)}{k_{m2} + x_0(t)} \\
\partial_t x_1(t) &= \frac{k_3 x_0(t) x_2(t)}{k_{m2} + x_0(t)} + k_5 x_2(t) - r_1(1-\eta) x_1(t) - d_3 \eta x_1(t) \\
\partial_t x_2(t) &= \frac{k_4}{b_2 + x_3(t)} - d_4 x_2(t) \\
\partial_t x_3(t) &= r_1(1-\eta) x_1(t) - k_5 x_3(t)
\end{aligned}
\tag{A6}
$$

with parameters given in the table below.

Variable	Name	Parameter Values
x_0	[IC]	$k_3 = 215$, $k_{m2} = 0.02$,
x_1	[BA]	$k_5 = 0.1$, $d_3 = 1$, $\eta = 0.05$, $r_1 = 0.1$
x_2	[C7H]	$k_4 = 1.169 \cdot 10^5$, $b_2 = 55$, $d_4 = 1.86$
x_3	[RBA]	

The variables are the concentrations of intercellular cholesterol [IC], bile acid [BA], Cholesterol 7α Hydroxylase [C7H], and Returned Bile Acids [RBA]. The 'external signal' is modeled by a uniform distribution, means $D \sim \text{Uniform}[0, 1]$.

Gene Regulatory Interactions in E. coli

As mentioned, we also examine a more biologically realistic model, which is based on a subset of an *E. coli* gene regulatory network and was provided in the DREAM challenge [3–5]. There, the *in-silico* network inference challenge investigated how well gene networks can be deduced from simulated data. The network is derived as subgraphs from the recognized gene regulation networks of *E. coli* and *S. cerevisiae* [6]. That is, the results presented are thus biologically realistic, i.e., aiming to capture a reasonable network but not given from a real observation, and the gene indexes are thus arbitrary. The gene regulatory network of

E. coli is defined and simulated with the following equations:

$$
\begin{aligned}
\partial_t x_1 &= \frac{k_{0,1}}{K_{M,1} + D} - \gamma_1 x_1 \\
\partial_t x_2 &= \frac{k_{0,2}}{K_{M,2} + x_1} + \frac{k_{0,3} x_3}{K_{M,3} + x_3} + \frac{k_{0,4} x_7}{K_{M,4} + x_7} - \gamma_2 x_2 \\
\partial_t x_3 &= \frac{k_{0,5} x_1}{K_{M,5} + x_1} + \frac{k_{0,6}}{K_{M,6} + x_2} + \frac{k_{0,7} x_5}{K_{M,7} + x_5} + \frac{k_{0,8} x_7}{K_{M,8} + x_7} - \gamma_3 x_3 \\
\partial_t x_4 &= \frac{k_{0,9}}{K_{M,9} + x_1} - \gamma_4 x_4 \\
\partial_t x_5 &= \frac{k_{0,10}}{K_{M,10} + x_2} - \gamma_5 x_5 \\
\partial_t x_6 &= \frac{k_{0,11} U}{K_{M,11} + U} - \gamma_6 x_6 \\
\partial_t x_7 &= \frac{k_{0,12} x_4}{K_{M,12} + x_4} + \frac{k_{0,13} x_6}{K_{M,13} + x_6} - \gamma_7 x_7
\end{aligned}
\tag{A7}
$$

with parameters given in the table below.

Gene	Parameter Values
N_1	$k_{0,1} = 0.0362$, $K_{M,1} = 0.1259$, $\gamma_1 = 0.4060$,
N_2	$k_{0,2} = 1.0106$, $K_{M,2} = 1.7937$, $k_{0,3} = 0.3550$, $K_{M,3} = 1.2069$, $k_{0,4} = 0.7472$, $K_{M,4} = 1.2858$, $\gamma_2 = 2.1362$
N_3	$k_{0,5} = 2.4007$, $K_{M,5} = 0.8218$, $k_{0,6} = 0.8511$, $K_{M,6} = 1.7099$, $k_{0,7} = 2.8247$, $K_{M,7} = 1.6656$, $k_{0,8} = 0.6081$, $K_{M,8} = 0.0202$, $\gamma_3 = 3.8740$,
N_4	$k_{0,9} = 0.0903$, $K_{M,9} = 0.069$, $\gamma_4 = 0.7256$
N_5	$k_{0,10} = 0.5264$, $K_{M,10} = 0.9600$, $\gamma_5 = 0.7466$
N_6	$k_{0,11} = 0.6541$, $K_{M,11} = 1.0891$, $\gamma_6 = 0.4525$
N_7	$k_{0,12} = 0.0090$, $K_{M,12} = 0.5191$, $k_{0,13} = 1.1236$, $K_{M,13} = 0.4986$, $\gamma_7 = 0.9473$

These parameters are taken from [3–5]. The 'external disturbance' is modeled by uniform distribution, means $D, U \sim \text{Uniform}[0, 1]$.

Sphingolipid Metabolism

The model includes reactions involved in sphingomyelin hydrolysis, the de novo synthesis of ceramide, and the salvage pathway, where we are specifically following three fatty acid chain molecular species. The specific reactions included in the model, as well as the enzymes included in the model, are shown in Figure 7 (C) in the main text. The abbreviations used in this figure are as follows. CER: Ceramide, HEXCER: Hexosylceramide, PC: Phosphatidylcholine, S1P: Sphinganine-1-phosphate, SM: Sphingomyelin, SPH: Sphinganine. The model aims to include the number of enzymes shown to be involved in these reactions (following reaction information obtained from KEGG: map00600, UNIPROT, and Rhea), and does not represent any specific biological situation. The enzyme kinetics are calculated using the method developed by Kroll et al. [7]. The kinetic rate for each enzyme involved in the reaction is calculated separately for specific reactant and product pairs, where the model uses the enzyme sequence and reactant and product SMILES strings to predict the kinetic rate. The mass action model of the reaction is calculated using the sum of the kinetic parameters for all enzymes involved in the reaction. The model is run until reaching the steady state where the values for serine, 3keto dihydrosphiganine, and PC are kept constant, as necessary metabolites for the system. For other metabolites, we assume a closed system. This model was simulated using MATLAB (MathWorks, Inc.). The MATLAB code for this model will be made available upon reasonable request.

Appendix B: Overview of Pairwise Orientation Methods

Several notable methods have been proposed to address the bivariate causal discovery problem. Early techniques relied on strong assumptions about the dynamic mechanisms, such as additive noise or functional models. However, biochemical variables may not meet these assumptions, particularly when they evolve within complex interaction networks. To determine which methods might be suitable for network orientation analyses, we examine various approaches using synthetic data.

The techniques we examined are based on three features: (1) statistical scores quantified for both directions and presume some statistical dependence (e.g., additional noise model), (2) modeling using neural networks, and (3) an ensemble of models using the concept of meta-learning. Briefly, the statistical scores require us to assume the dynamic mechanism between the two variables but allow inference from a relatively low number of data points. Conversely, methods that involve stacking of statistical features or neural network modeling are heavily data-consuming.

1. Statistical Scores

Methods for interaction direction inference that involve some statistical scores aim to quantify the asymmetric conditional dependence between the two variables. However, these statistical scores are strongly based on functional causal models (FCM) between the two continuous variables. It means that for two variables x and y, the edge $x \rightarrow y$ means that $y = f(x, \theta, \eta)$ where θ is the parameter set and η is a noise term. The causal structure is identifiable whenever there are no unobserved confounders, it belongs to a restricted functional class, and suitable constraints are imposed on η. It has been shown that without any further assumption on the function f, the causal direction is not identifiable because for both directions one can find an independent noise term [8, 9]. Inspired by [10], we test the following statistical scores in the benchmark.

Additive Noise Model (ANM)

The additive noise model (ANM) assumes that $y = f(x) + \eta$ where noise η and the variable x are independent. The additive noise model is one of the most popular approaches

for pairwise causality. It is based on the fitness of the data to the additive noise model in one direction and the rejection of the model in the other direction. The data is assumed to be continuous [11].

Conditional Distribution Similarity statistics (CDS)

Assume that the shape of the conditional distribution $p(Y|X = x)$ tends to be similar for different values of x if the random variable X is the cause of Y (i.e., if $X \rightarrow Y$). Then, one of the quantities that captures this variability is the standard deviation of the scaled values of y after binning in the x direction. A lower standard deviation indicates $x \rightarrow y$. This measure is defined as $CDS = \frac{1}{M} \sum_{y=0}^{M-1} \mathrm{var}_x[P_{\mathrm{normalized}}(y|x)]$ (see [12]).

Information Geometric Causal Inference (IGCI)

This approach considers a deterministic process that follows $y = f(x)$, where the function f is assumed to be invertible, which means that f^{-1} exists. The direction of interaction is then determined by measuring the irregularities by the distance to an exponential family using the information statistics metric, i.e., Kullback-Leibler statistics [13]. Furthermore, the authors show the applicability of such statistical measures for processes evolving with small additive noise [13].

Regression Error based Causal Inference (RECI)

The approach is based on the assumption that a regression fit in the true causal direction yields smaller errors, on average, than when fitting the model in the opposite direction. It allows non-deterministic nonlinear relations between the cause and the effect. The analysis takes place by fitting a least squares regression in both possible causal directions, and the causal direction is chosen to be the direction with the lowest mean-squared errors (MSE) [14]. For regression implementations, we use polynomial regression of degree 3 [15].

2. Machine Learning Techniques

Beyond the statistical methods presented above, which, as mentioned, require some strong assumptions or prior knowledge about the causal mechanism, different approaches use machine learning (ML) techniques to detect the direction of the interaction. These methods, however, pose some challenges, since they require prior learning of a labeled training set and require large datasets that are not necessarily accessible. Moreover, these methods require additional cost, the need for graphics processing units (GPUs), and computational time due to the complexity of the algorithms.

Generally, ML methods aim to infer the direction of interaction from hidden patterns within the training data, without explicitly coding the features required for inference. It can be done by using neural network (NN) modeling, or by stacking many properties of the system in the so-called meta-learning techniques.

Causal Generative Neural Networks (CGNN)

The method aims to learn the multivariate function causal model f using a generative NN. The uses of NN to learn f allow for not explicitly restricting the class of functions allowed. Particularly, it models f for both directions, $y \rightarrow x$ and $x \rightarrow y$, with a neural network with one hidden layer. Then it chooses the direction that provides the best fit - using a non-parametric score, the Maximum Mean Discrepancy [16].

Randomize Causation Coefficient (RCC)

The method utilizes a NN that is constructed with two parts - kernel embedding layers and a classifier. The former part is based on the projection of the observational distributions into Reproducing Kernel Hilbert Space (RKHS) using random cosine embedding, and the classifier is a random forest [17].

Neural Causation Coefficient (NCC)

The neural network (NN) is designed with embedding layers that facilitate the learning of feature maps, which are essential for understanding the data. Following the embedding

layers, the architecture includes binary classifier layers, both of which are structured as multilayer perceptrons [18].

Stacking with Gradient Boosting Classification - JARFo

This method is an ensemble learning algorithm of many statistical features, including statistical measures, information measures, and measures of the conditional probability variability. The stacking of all these features was done with gradient-boosting classification. In the literature, it is named after its designer - José A. R. Fonollosa (JARFo) - [12].

Causal Ensemble Measure Machine (CEMM)

Stacking the statistical scores using the support measure machine (SMM) as follows. First, train SMM, which classifies each score as to whether it successfully detects the direction or not. The stacking involves 'flipping' the wrongly assigned direction [10].

Appendix C: Imputation

In Fig. 3 of the main text, we present results for different inference methods as the number of data points N is varied. This variation in N is equivalent to removing observations with missing values from the dataset, thus reducing the number of available data points. Alternatively, imputation strategies can also be considered. Fig. A shows the accuracy of directional inference methods under several such approaches. Mean imputation replaces missing values in a dataset with the mean of the observed values for that variable. The k-nearest neighbors (KNN) imputation method estimates missing values using information from the k most similar data points, while MICE (Multiple Imputation by Chained Equations) iteratively predicts missing values from other variables in the dataset. The results obtained by varying the missing data rate are consistent with those presented in Fig. 3 of the main text when varying N.

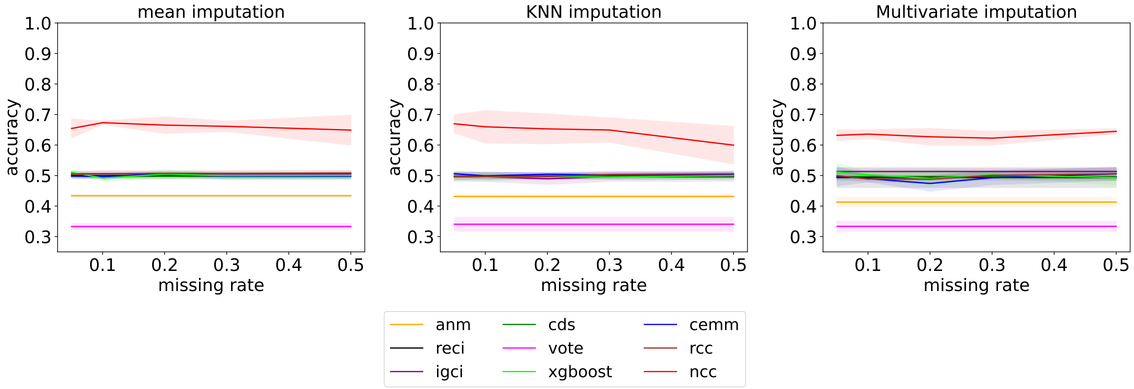

FIG. A. Accuracy of directional inference methods under different data imputation strategies. Mean imputation method fills the missing data with their corresponding average. The KNN method estimates missing values based on the $k = 5$ most similar data points in the feature space, and the multivariate imputation uses iterative approach. Results are shown for Michaelis–Menten dynamics with a noise scale of 0.5 and a dataset size of $N = 10^3$. The missing rates vary between 0.05 to 0.5.

[1] M. Nitzan, J. Casadiego, and M. Timme, Revealing physical interaction networks from statistics of collective dynamics, Science advances **3**, e1600396 (2017).

[2] J. Casadiego, M. Nitzan, S. Hallerberg, and M. Timme, Model-free inference of direct network interactions from nonlinear collective dynamics, Nature communications **8**, 2192 (2017).

[3] T. Schaffter, D. Marbach, and D. Floreano, Genenetweaver: in silico benchmark generation and performance profiling of network inference methods, Bioinformatics **27**, 2263 (2011).

[4] F. Liu, S.-W. Zhang, W.-F. Guo, Z.-G. Wei, and L. Chen, Inference of gene regulatory network based on local bayesian networks, PLoS computational biology **12**, e1005024 (2016).

[5] M. Foo, J. Kim, and D. G. Bates, Modelling and control of gene regulatory networks for perturbation mitigation, IEEE/ACM Transactions on Computational Biology and Bioinformatics **16**, 583 (2018).

[6] D. Marbach, T. Schaffter, C. Mattiussi, and D. Floreano, Generating realistic in silico gene networks for performance assessment of reverse engineering methods, Journal of computational biology **16**, 229 (2009).

[7] A. Kroll, S. Ranjan, M. K. Engqvist, and M. J. Lercher, A general model to predict small molecule substrates of enzymes based on machine and deep learning, Nature communications **14**, 2787 (2023).

[8] A. Hyvärinen and P. Pajunen, Nonlinear independent component analysis: Existence and uniqueness results, Neural networks **12**, 429 (1999).

[9] K. Zhang, Z. Wang, J. Zhang, and B. Schölkopf, On estimation of functional causal models: general results and application to the post-nonlinear causal model, ACM Transactions on Intelligent Systems and Technology (TIST) **7**, 1 (2015).

[10] G. Varando, S. Catsis, E. Diaz, and G. Camps-Valls, Pairwise causal discovery with support measure machines, Applied Soft Computing **150**, 111030 (2024).

[11] P. Hoyer, D. Janzing, J. M. Mooij, J. Peters, and B. Schölkopf, Nonlinear causal discovery with additive noise models, Advances in neural information processing systems **21** (2008).

[12] J. A. Fonollosa, Conditional distribution variability measures for causality detection, Cause Effect Pairs in Machine Learning , 339 (2019).

[13] Inferring deterministic causal relations, arXiv preprint arXiv:1203.3475 (2012).

[14] P. Blöbaum, D. Janzing, T. Washio, S. Shimizu, and B. Schölkopf, Analysis of cause-effect inference by comparing regression errors, PeerJ Computer Science **5**, e169 (2019).

[15] D. Kalainathan, O. Goudet, and R. Dutta, Causal discovery toolbox: Uncovering causal relationships in python, Journal of Machine Learning Research **21**, 1 (2020).

[16] O. Goudet, D. Kalainathan, P. Caillou, I. Guyon, D. Lopez-Paz, and M. Sebag, Learning functional causal models with generative neural networks, Explainable and interpretable models in computer vision and machine learning , 39 (2018).

[17] D. Lopez-Paz, K. Muandet, B. Schölkopf, and I. Tolstikhin, Towards a learning theory of cause-effect inference, in *International Conference on Machine Learning* (PMLR, 2015) pp. 1452–1461.

[18] D. Lopez-Paz, R. Nishihara, S. Chintala, B. Scholkopf, and L. Bottou, Discovering causal signals in images, in *Proceedings of the IEEE conference on computer vision and pattern recognition* (2017) pp. 6979–6987.
